# Characterization of flagellins isolated from a highly motile strain of *Lactobacillus agilis*

**DOI:** 10.1186/s12866-016-0667-x

**Published:** 2016-03-22

**Authors:** Akinobu Kajikawa, Emiko Midorikawa, Kazuya Masuda, Kazuho Kondo, Tomohiro Irisawa, Shizunobu Igimi, Sanae Okada

**Affiliations:** Department of Applied Biology and Chemistry, Tokyo University of Agriculture, 1-1-1 Sakuragaoka, Setagaya, Tokyo 156-8502 Japan; Division of Biomedical Food Research, National Institute of Health Sciences, 1-18-1 Kamiyoga, Setagaya, Tokyo 158-8501 Japan; Department of Animal Science, Tokyo University of Agriculture, 1737 Funako, Atsugi, Kanagawa 243-0034 Japan

## Abstract

**Background:**

Most lactic acid bacteria are non-motile but some of them are flagellated and exhibit motility. So far, motile lactobacilli have rarely been studied, and characteristics of their flagellins are poorly understood. In this study, a highly motile strain of *Lactobacillus agilis* was recruited for transcriptional analysis and characterization of its flagellins.

**Results:**

Unlike another motile lactic acid bacteria of intestinal isolate, *Lactobacillus ruminis*, flagellar filaments of the *L. agilis* strain probably consist of two homologous but distinct flagellins. Glycosylation of the flagellar filaments and their resistance to heat, acid and SDS were also observed. The immunological activity of the flagellins was evaluated through the stimulation of Caco-2 cells. The results show that TLR5-stimulating activity of the protein is attenuated, likely due to an incomplete TLR5-recognition site.

**Conclusions:**

The flagella filaments of *L. agilis* BKN88 consist of two homologous glycosylated flagellins, which likely have an incomplete TLR5-recognition site. The characteristics of the flagellin are presumably a consequence of adaptation as a commensal microbe in the gastrointestinal tract.

## Background

While lactic acid bacteria are generally non-motile, some of them are flagellated and exhibit motility. *Lactobacillus agilis* and *Lactobacillus ruminis* are representatives of those flagellated microbes which have been primarily isolated from homeothermal animals such as cows, pigs, birds, etc. [1–5]. So far, a few studies on these lactobacilli have been done, but their characteristics are barely understood. Flagella have a complex protein structure and their filaments are made up of a subunit protein referred to as flagellin. Flagellins are also known as ligands of Toll-like receptor 5 (TLR5), one of pattern recognition receptors [6]. In a previous study, Neville et al. presented the genetic and transcriptional analysis of the motility-associated genes of *L. ruminis*. The immunological activity of flagellin isolated from *L. ruminis* and a few other flagellated lactobacilli via TLR5 was also reported, although flagellins of *L. agilis* were not included [7].

Our group has recruited *L. agilis* as a model of motile lactobacilli which reside in the gastrointestinal mucosa. Although *L. agilis* is frequently isolated from birds, there are other isolates from pigs and humans [8, 9]. In addition, we isolated a few *L. agilis* strains from lemurs and tapirs (unpublished). Hence, *L. agilis* may be considered as a commensal distributed among various animals. A major advantage in the study of this species is that it is capable of transformation with well-established plasmid vectors such as pGK12 and derivatives [10] (our unpublished data). Thus *L agilis* can be investigated using genetic manipulations or applied as biotherapeutics, such as vaccine/drug-delivery systems [11]. While *L. agilis* appears to be a promising model, however, it has not been characterized sufficiently.

This study focuses on the flagellins of a highly motile strain, *L. agilis* BKN88, which was derived from a chicken isolate strain, JCM 1048. Firstly, the expression profile of the two copies of flagellin-encoding genes was determined. Secondly, posttranscriptional modification of the proteins was shown, and the stability of the flagellar filaments under depolymerizing conditions was tested. Lastly, the immunological activity of the flagellins was compared to that of the orthologous proteins of pathogenic bacteria in their interaction with Caco-2 cells. The aim of this study is to reveal previously unknown characteristics of flagellins of the motile commensal *L. agilis*.

## Results

### Motility of *L. agilis* strains

**Fig. 1:**
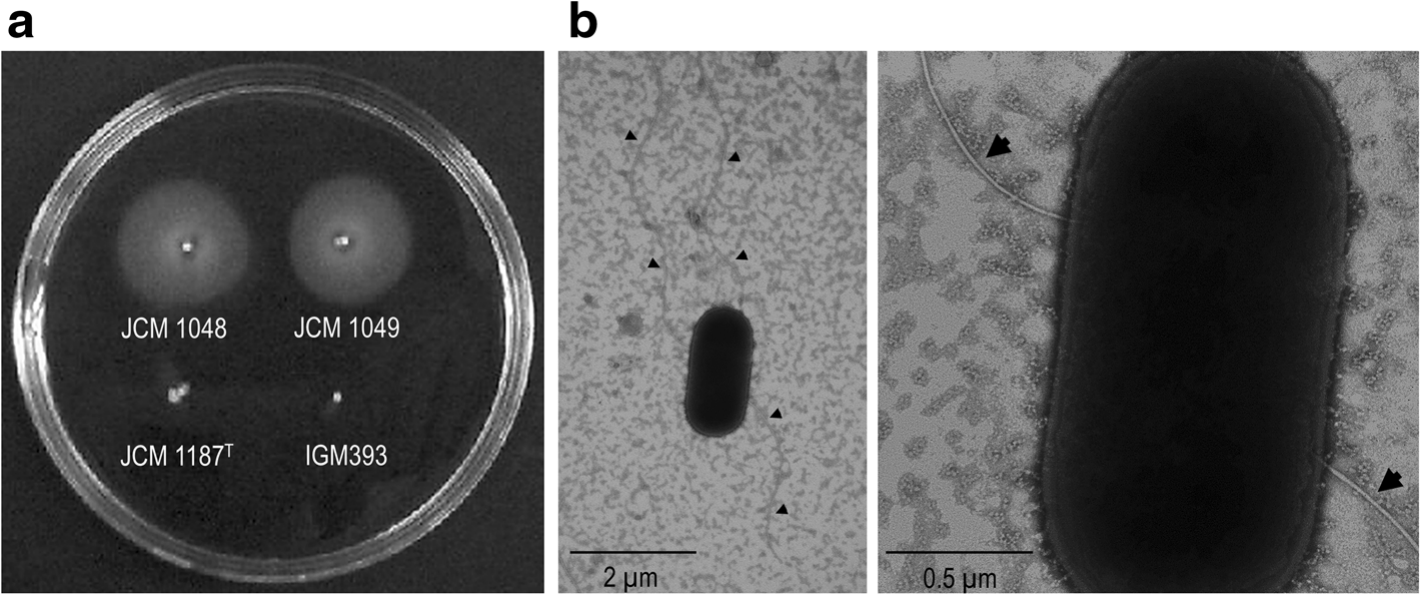
Observation of motility and flagella. **a** The motility of *L. agilis* strains was determined using semi-solid MRS-agar medium. Three *L. agilis* strains and *L. paracasei* IGM393 (non-motile) were stabbed and incubated overnight. **b** TEM image of *L. agilis* BKN88 grown on an MRS-agar plate. The rates of magnification were 13900:1 (*left*) and 76600:1 (*right*). Arrow heads point flagellar filaments of BKN88

The motility of each *L. agilis* strain was confirmed in MRS soft-agar culture. JCM 1048 and JCM 1049 spread in the semi-solid medium while JCM 1187^T^ and *L. paracasei* IGM393 did not (Fig. 1a). The motile strains were then observed by microscopy; however, only a small fraction of the cells were actively swimming. In order to isolate a uniformly motile cell culture, hundreds of subcultures of JCM 1048 and JCM 1049 were investigated. With this screening, a highly motile subculture of JCM 1048, identified as BKN88, was obtained. In the culture of BKN88, almost all cells were actively moving at exponential phase, with decreasing motility at late exponential phase to stationary phase. TEM analysis showed multiple filaments around the BKN88 cells, indicating peritrichous flagella of the bacterium.

### Expression of flagellin genes

**Fig. 2:**
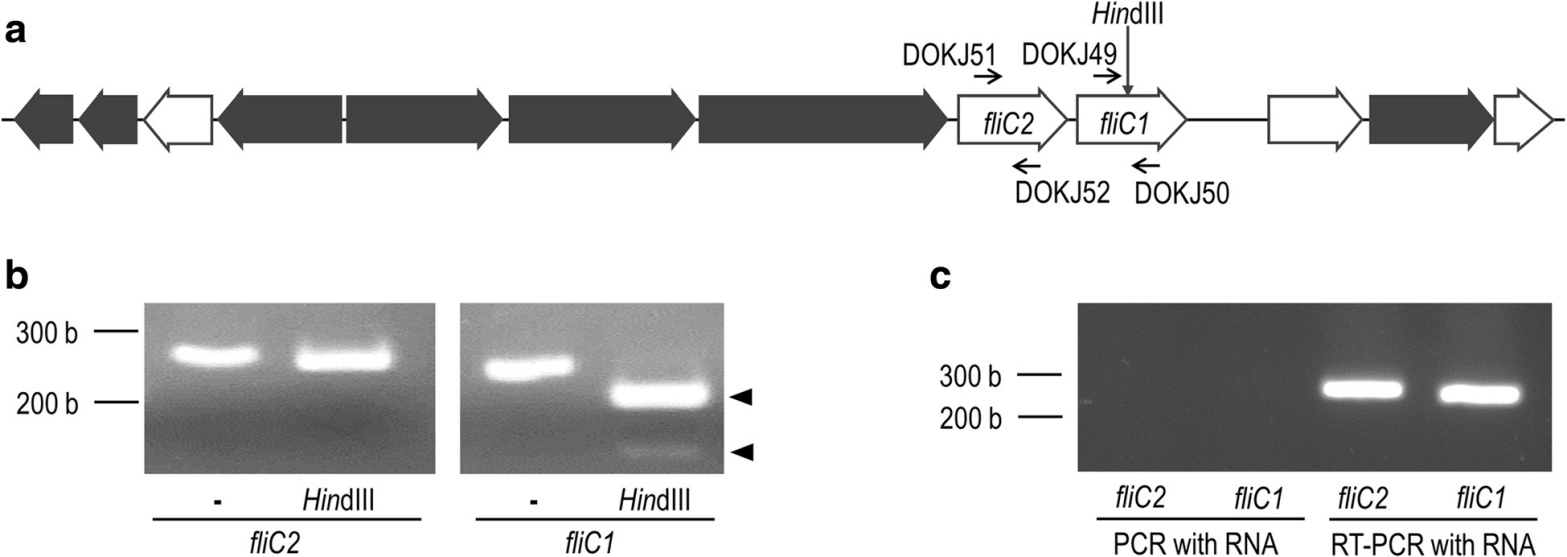
Genetic analysis of the flagellin-encoding genes of *L. agilis* BKN88. **a** Partial gene map of the *L. agilis* motility operon. PCR amplification primers and the *Hin*dIII site are shown. Filled arrows represent putative glycosyltransferases. **b** Validation of flagellin-specific primers. Partial fragments of *fliC1* and *fliC2* were amplified by PCR using the specific primers and digested with *Hin*dIII. **c** Transcription of fliC1/fliC2 was detected by RT-PCR. PCR without reverse transcription was also performed to detect contaminated DNA

Based on the sequence of *L. agilis* DSM 20509^T^, which has recently been deposited to GenBank, a partial gene map of the motility operon is shown in Fig. 2a. Two flagellin-encoding genes with 90 % homology of both their nucleotides and amino acids are located in tandem. To discriminate the transcription of the two homologous flagellin-encoding genes, *fliC1* and *fliC2*, specific primers were designed. Since only the amplicon of *fliC1* includes a HindIII site, the specificity of the primers were easily validated by digestion of the PCR products with the restriction enzyme (Fig. 2b). RT-PCR to detect transcription of *fliC1*/*fliC2* in BKN88 was performed at early exponential phase, when the culture showed the highest motility. As shown in Fig. 2c, expression of both *fliC1* and *fliC2* were detected.

### Composition and posttranslational modification of the flagellins

**Fig. 3:**
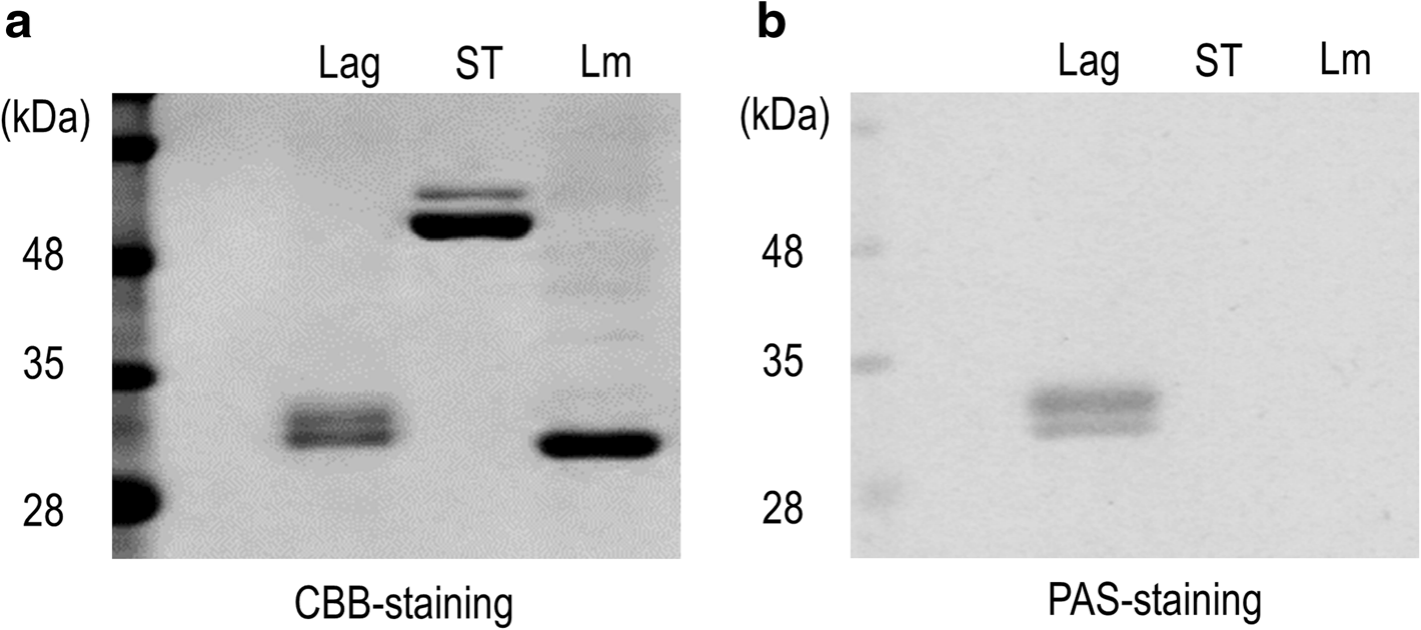
SDS-PAGE and staining. Isolated flagellins from *L. agilis* BKN88 (Lag), *S.* Typhimurium 92–35 (ST), and *L. monocytogenes* EGD (Lm) were separated by SDS-PAGE and stained with CBB (**a**) or PAS (**b**). The molecular mass of the standard marker bands are shown at the left margin

**Fig. 4:**
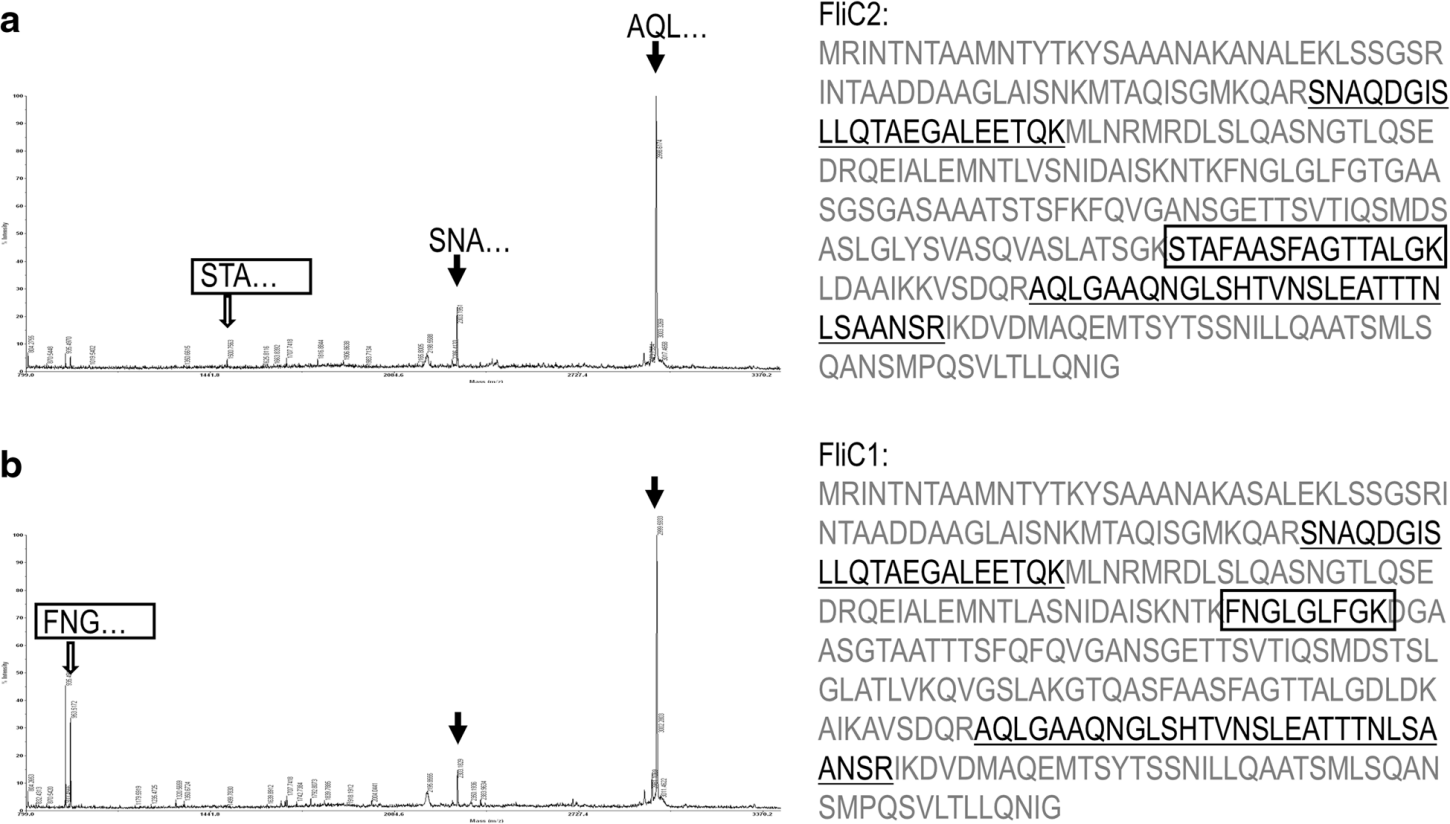
Identification of flagellins by MALDI-TOF MS analysis. Protein bands were separately collected from SDS-PAGE gels and analyzed. Mass spectra of the upper band (**a**) and lower band (**b**) are shown. Filled arrows represent signals consistent with the mass of underlined peptides. Open arrows represent signals consistent with boxed peptides

Flagellar filaments of BKN88, *L. monocytogenes* EGD, and *S.* Typhimurium 92–35 were isolated by ultracentrifugation. The isolated proteins were then analyzed by SDS-PAGE and CBB staining. As shown in Fig. 3a, double bands which have slightly different molecular mass from each other were detected from BKN88. To identify these two proteins, each band was collected separately and analyzed by MALDI-TOF MS after digestion with trypsin. The mass spectra obtained from the two protein bands appeared to be similar, with minor differences (Fig. 4). Although the overall coverage of the peptide mass fingerprinting was low, two major peak of both mass spectra were consistent with those of peptides from FliC1 (the lower band) and FliC2 (the upper band). Since there are multiple glycosyltransrerase genes at flanking region of the flagellin genes (Fig. 2a), glycosylation of the flagellins was predicted. PAS-staining of acrylamide gels indicated that both FliC1 and FliC2 underwent posttranslational modification (Fig. 3b). Neither the flagellins of *S.* Typhimurium nor those of *L. monocytogenes* were stained with PAS as already known, albeit the flagellin of *L. monocytogenes* was glycosylated with N-acetylglucosamine [12].

### Depolymerization of flagellar filaments

**Fig. 5:**
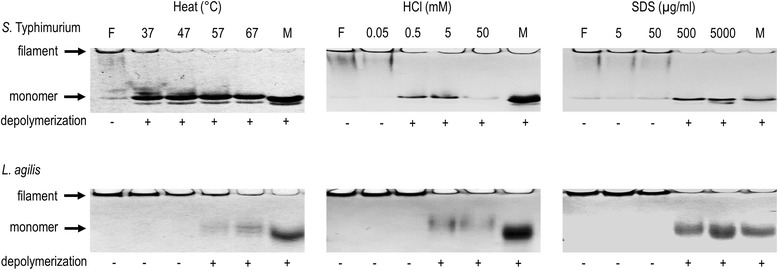
Stability of flagellar filaments in different conditions. Treated or non-treated flagellar filaments of *S.* Typhimurium 92–35 (*top*) and *L. agilis* BKN88 (*bottom*) were analyzed by Native-PAGE. Temperatures and concentrations of reagents are shown on the top margin. Occurrence of depolymerization was shown on the bottom margin. F: filament state, M: monomeric state

The stability of the flagellar filaments of *L. agilis* BKN88 and *S.* Typhimurium 92–35 were assessed by heating, and treatment with acid and detergent. After the purified flagellar filaments were incubated under the each condition, the proteins were applied to Native-PAGE gels. Since flagellar filaments are macromolecules, only the depolymerized proteins can travel in the gel. As shown in Fig. 5, the flagellar filaments of *L. agilis* BKN88 were more resistant to thermal or acidic conditions than those of *S.* Typhimurium 92–35. While the flagella of *S.* Typhimurium were depolymerized even at 37 °C, those of *L. agilis* were partially disassembled only at 57 °C. Monomeric flagellins of *L. agilis* were detected after treatment with 5 mM HCl; however, 0.5 mM HCl was sufficient to depolymerize the flagellar filaments of *S.* Typhimurium. Differences between the flagella of the two organisms were not observed under SDS-treatment.

### Immunological activity of the flagellins

**Fig. 6:**
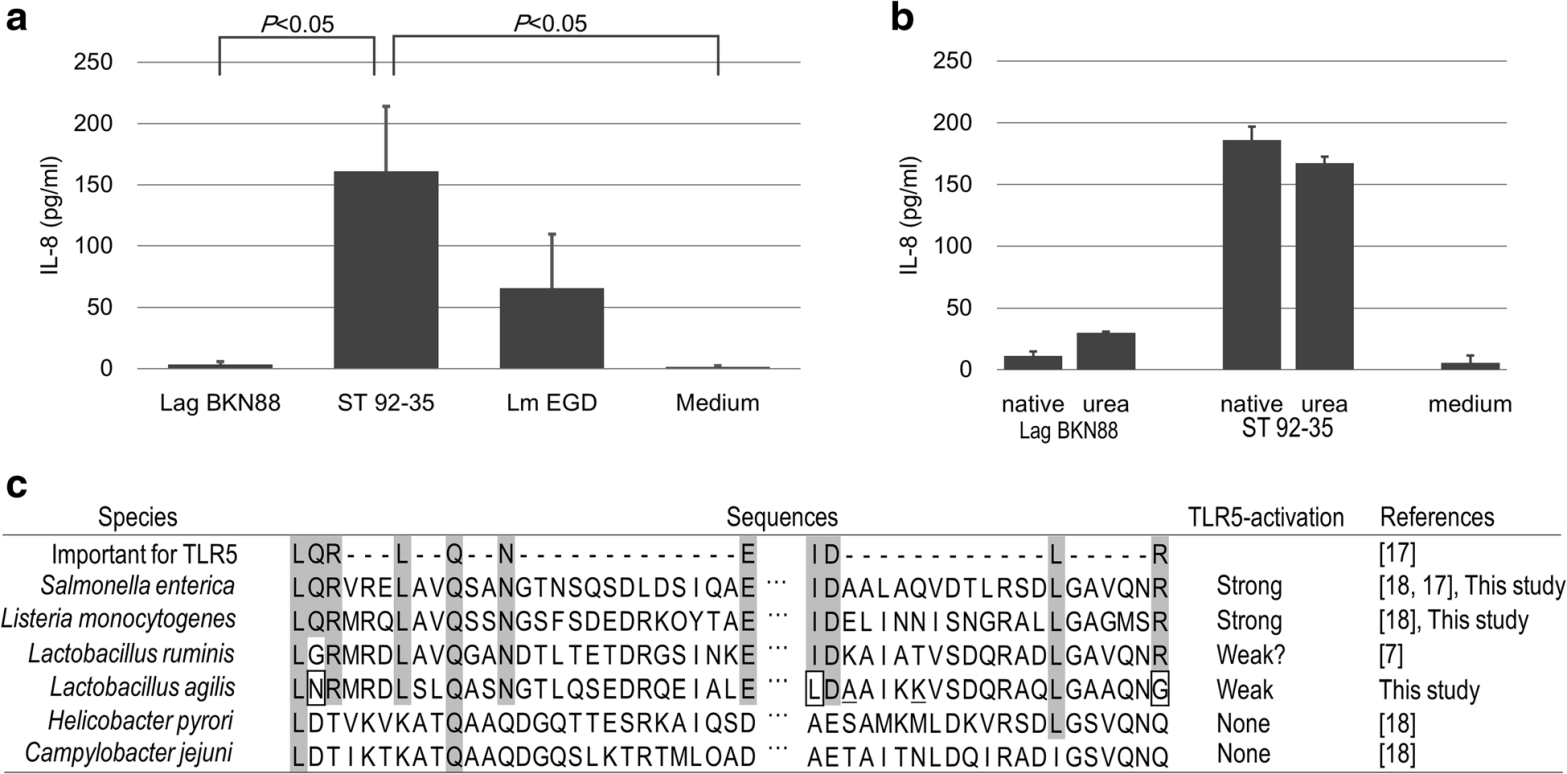
Analysis of the immunological properties of isolated flagellins. **a** Filamentous flagellins (10 pmol/ml) were added to Caco-2 cell cultures and released IL-8 was quantified by ELISA. Values are mean + SE (*n* = 4). Statistical significance was also shown (*P* < 0.05). **b** Caco-2 cells were stimulated with native (filamentous) or urea-treated (depolymerized) flagellins at 100 pmol/ml. Values are mean + SE (*n* = 3). **c** Alignment of amino acid sequences of major flagellins from pathogenic bacteria and lactobacilli (FliC2). Conserved residues which were reported to be important for TLR5 recognition by Smith et. al. are highlighted. Boxed letters are different from the conserved amino acid residues. Underlines represent polymorphic amino acids between FliC1 and FliC2: A and K are K and A in FliC1. References: [7, 17, 18]

IL-8 production from Caco-2 cells induced by stimulation with flagella/flagellins was assessed. With native flagella (filaments) of *L. agilis* BKN88, no clear IL-8 production was detected, while high levels of IL-8 was observed by stimulation with those of *S.* Typhimurium 92–35 and *L. monocytogenes* EGD (Fig. 6a). The same assay was performed with depolymerized flagellins prepared by chaotropic denaturation. After this treatments, the IL-8 response was slightly increased in *L. agilis* flagellins, but still much lower than that induced by *S.* Typhimurium (Fig. 6b). The conserved amino acid residues of bacterial flagellins which are critical for TLR5-recognition of *L. agilis* and other bacteria were compared (Fig. 6c). Most of the amino acid residues were conserved in the lactobacilli; however, there were a few differences between the commensal bacteria and the TLR5-stimulating pathogens. While *S.* Typhimurium and *L. monocytogenes* have -LQR- in the TLR5-recognition site, *L. ruminis* and *L. agilis* instead have -LGR- and -LNR- respectively. Moreover, two other differences at the site were found in *L. agilis* flagellins.

## Discussion

Motile lactobacilli have rarely been studied, and as commensals, the significance of their flagella remains unknown. With increasing interest in the immunological properties of lactic acid bacteria, the unique ability of the flagella of *L. agilis* to interact with TLR5 is of particular note. In this study, flagellins of *L. agilis* BKN88 were analyzed and some of their unique characteristics have been uncovered.

The flagellar filaments of *L. agilis* BKN88 consist of two homologous proteins, FliC1 and FliC2. This was confirmed at both the mRNA and protein level, although the coverage of peptide mass fingerprinting was low, possibly due to glycosylation of the flagellins which affects the mass of peptides. Previously, Neville et al. reported that a motile strain of *L. ruminis* ATCC 27782, which also has two flagellin genes, expressed *fliC2* dominantly and *fliC1* only slightly [7]. These facts indicate that flagellin-gene expression and composition of flagellins in the filaments are not uniform among motile lactobacilli.

PAS-staining showed that both the FliC1 and FliC2 flagellins were glycosylated. This posttranscriptional modification was previously reported in other bacteria and archaea [13, 14]. In those studies, glycosylation of flagellins was reported to be involved in stability, assembly, virulence, or motility of flagella or the flagellated microbes, albeit the functions are not fully understood. Types of molecular bonds between a flagellin and a glycan are either N- or O-linkage. Since only O-linkage bonds are found in Gram-positive bacteria, it is likely that lactobacilli have the same type of bond. A previous study showed that glycosylated flagellins of *Pseudomonas* strains were more immunologically active than unmodified flagellins [15]. Hence, the glycosylation of *Lactobacillus* flagellins might have an impact on their immunological properties. Further studies need to be done to investigate mechanisms and influences of the modification.

The flagellar filaments of *L. agilis* were relatively stable under acidic conditions. This characteristic seems to be reasonable because lactobacilli are routinely producers of lactic acid. The flagellar polymeric structure is found to be thermostable compared to that of *Salmonella*. Collectively, the flagella of *L. agilis* may be durable, which would support sustainable motility of the microbe. Resistance to depolymerization also suggests that the flagella are immunologically inactive, since TLR5 interacts with only monomeric flagellin but not with polymeric states [16, 17]. Indeed, the native flagella of *L. agilis* barely induced IL-8 production by Caco-2 cells while those of *S.* Typhimurium elicited the response due to their depolymerization at 37 °C. This might contribute to the symbiotic relationship between *L. agilis* and its host, by avoiding undesired immunostimulation.

As previously reported, the flagellins of *L. ruminis* present their immunological activity via interaction with TLR5 [7]. Likewise, monomeric flagellins of *L. agilis* exhibit the proinflammatory characteristic; however, the activity was found to be much lower than that of *S.* Typhimurium and *L. monocytogenes* in this study. Previously, specific amino acid residues conserved among the D1 domain of *Salmonella* FliC were proven to be critical for TLR5-recognition [17]. The independent study by Verma et al. also demonstrated that replacement of amino acids at this conserved site of the *Pseudomonas aeruginosa* flagellin drastically reduced IL-8 response via TLR5 [15]. *H. pylori* and *C. jejuni* replaced most of those amino acid residues in their flagellins and thus lack TLR5-stimulating activity. Furthermore, a chimeric *Salmonella* FliC in which the TLR5-recognition site was replaced with the corresponding site of flagellin from *H. pylori* failed to interact with TLR5, which supports the importance of those amino acid residues [18]. In *Lactobacillus* flagellins, a few mismatchs in those sequences conserved among high TLR5-activators such as the flagellins of *S.* Typhimurium and *L. monocytogenes* were found. Hence, it is likely that the attenuated immunological activity of *L. agilis* flagellins are attributed to the slight but influential differences at the TLR5-recognition site. As a symbiotic bacterium, *L. agilis* may not be involved in proinflammatory responses at the gastrointestinal mucosa. Presumably, the attenuation on the TLR5 ligand could help the bacterium to avoid exclusion by the local immune system of the hosts.

## Conclusions

The flagella filaments of *L. agilis* BKN88 consist of two homologous glycosylated flagellins and are relatively stable in acidic and thermal conditions. The flagellins likely have an incomplete TLR5-recognition site, which may be the reason for their attenuated immunological activity. These characteristics of the flagellin are presumably a consequence of adaptation as a commensal microbe.

## Methods

### Bacterial strains and growth conditions

*Lactobacillus paracasei* IGM393 (former ATCC393, laboratory strain) [19]*, Lactobacillus agilis* JCM 1048 (avian isolate), JCM 1049 (porcine isolate), JCM 1187^T^ (swage water isolate), and BKN88, a highly motile variant of JCM 1048, were used in this study. All JCM strains were purchased from RIKEN BioResource Center, Ibaraki, Japan. *L. agilis* strains were grown statically (liquid culture) or anaerobically (plate culture) using AnaeroPouch-Anaero Anerobic Gas Generators (Mitsubishi Gas Chemical) in MRS broth/agar (BD) at 37 °C. Motilities of lactobacillus strains were determined by inoculation into semi-solid MRS medium with 0.2 % agar. Bacterial motility was also observed using an optical microscope. *Salmonella enterica* subsp. *enterica* serovar Typhimurium 92–35 (laboratory stock) was grown aerobically (shaking) in Brain Heart Infusion (BHI) broth (BD) at 37 °C. *Listeria monocytogenes* EGD [20] was grown statically in BHI broth at 25 °C.

### Transmission electron microscopy (TEM)

Bacterial cells prepared from colonies on a MRS-agar plate were absorbed to formvar film coated copper grids (400 mesh) and stained with 2 % phosphotungstic acid solution (pH 7.0). The bacterial cells and the flagellar filaments with negative staining were visualized using a transmission electron microscope (JEM-1400Plus, JEOL Ltd., Tokyo, Japan) at an acceleration voltage of 80 kV. Digital images were taken with a CCD camera (VELETA, Olympus Soft Imaging Solutions GmbH, Münster, Germany).

### PCR and reverse transcriptional PCR (RT-PCR)

Specific primers for detection of each flagellin-encoding gene (*fliC1* and *fliC2*) were designed in reference to the sequence of the *L. agilis* DSM 20509^T^ motility operon (GenBank, Acession# KM886859). Primer pairs, DOKJ49 (CTT TGG AAA AGA TGG TG) and DOKJ50 (CAG CCT TGA TAG CTT T) for *fliC1*, DOKJ51 (TTT CGG TAC AGG TGC A) and DOKJ52 (CTT TCT TGA TAG CAG C) for *fliC2*, were used for PCR. Total RNA was prepared from bacterial cultures at early exponential phase. Fresh MRS broth was inoculated with a 1 % volume of overnight culture of *L. agilis* BKN88 and incubated until the optical density (600 nm) reached 0.2. Cells were collected and washed twice with TE buffer, followed by bead beating with a FastPrep Instrument (MP Biomedicals) in RNAprotect Bacteria Reagent (QIAGEN). The bacterial RNA was purified using an RNeasy Mini Kit (QIAGEN) in accordance with the manufacturer’s instruction. RT-PCR was performed using a PrimeScript One Step RT-PCR kit (Takara). Contamination of DNA in the RNA sample was tested for using Ex. *taq* DNA polymerase (Takara).

### Isolation of flagellar filaments and monomeric flagellins

Flagellar filaments were isolated in accordance with protocols described elsewhere [21]. Briefly, bacterial cells at mid exponential phase (OD600 of 1.0) were collected and vortexed intensively in distilled water. Cells were then removed by centrifugation at 8,000 × g for 5 min. The cell free supernatants were then fractionated by ultracentrifugation. Pellets were suspended in a small amount of distilled water and stored at −20 °C until use. For preparation of monomeric flagellins, the purified flagellar filaments were incubated in 6 M urea solution and diluted with the medium used for Caco-2 cell culture (described below).

### SDS-PAGE and staining

Protein samples were prepared by mixing with an equal volume of 2× Laemmli buffer containing 5 % beta-mercaptoethanol and boiled for 5 min. Proteins were separated in 5–20 % polyacrylamide gradient c-PAGEL (ATTO) gels by SDS-PAGE. Protein bands were visualized by either staining with Quick-CBB PLUS (Wako) or periodic acid Schiff (PAS) staining. For PAS staining, gels were pretreated with 40 % methanol and 7 % acetic acid for 1 h. After 1 h incubation with 7.5 % acetic acid, gels were treated with 1 % periodic acid for 1 h and washed intensively in 7.5 % acetic acid. Glycoproteins were then stained with Schiff’s reagent for 1 h and washed with 0.5 % sodium bisulfite solution.

### Matrix Assisted Laser Desorption/Ionization time-of-flight mass spectrometry (MALDI-TOF MS)

Protein bands were enzymatically digested in-gel by treatment with porcine trypsin as described previously (Shevchenko et al. Anal. chem. 1996, 68:850–858). Briefly, gel pieces were washed with 50 % acetonitrile to remove SDS, salt and CBB stain. Washed and dehydrated spots were then vacuum dried to remove solvent and rehydrated with trypsin (8–10 ng/μl) solution in 50 mM ammonium bicarbonate pH 8.7 and incubated 8–10 h at 37 °C. Samples were analyzed using the Applied Biosystems 4700 proteomics analyzer with TOF/TOF™ ion optics. MS data was acquired with a Nd: YAG laser with 200 Hz repetition rate, and up to 4000 shots were accumulated for each spectrum. MS data was acquired using the instrument default calibration, without applying internal or external calibration. These procedures were done by Genomine, Inc. (Korea).

### Flagella depolymerization assay

Depolymerization of flagellar filaments were assessed by heating at 37-67 °C, incubation in 0.05-50 mM HCl solution at room temperature, or treatment with 5–5000 μg/ml of SDS at room temperature. After the treatments for 10 min, HCl was neutralized by adding 0.5 M of NaHCO_3_ solution for 50 mM of HCl or 50 mM of Tris-Cl (pH 8.0) for lower HCl concentrations. The treated samples were mixed with equal volumes of 2× Native-PAGE sample buffer (30 % glycerol, 1 % bromophenol blue, 125 mM Tris–HCl, pH 6.8). Samples were then loaded to c-PAGEL compact gels (ATTO) and electrophoresed using Tris-Glycine buffer (BIO-RAD). After 30 min of electrophoresis (Native-PAGE), the gels were stained with CBB. As references, non-treated flagella mixed with Native-PAGE sample buffer (filament) and the protein mixed with Laemmli buffer containing β-melcaptoethanol (monomeric flagellin) were also prepared.

### Caco-2 cell-stimulation assay

Caco-2 cells, originating from the human colon, were maintained in Dulbecco’s modified Eagle Medium (DMEM, Wako) supplemented with 10 % fetal bovine serum (BD), non-essential amino acids, glutamax (Thermo Fisher Scientific), and penicillin/streptomycin in 5 % CO_2_ at 37 °C. Semi-confluent cultures of Caco-2 cells were collected by treatment with Tripsisn-EDTA solution (Life Technologies) and washed with the medium. The cells were then seeded to 96-well flat-bottom microplate wells (Thermo Fisher Scientific) at a concentration of 2x10^4^ cells/well. Flagellin solution (100 pmol/ml) was added to each well and incubated for 4 h. Culture supernatants were then collected and stored at −20 °C until use. Human interleukin-8 (IL-8) was quantified with the OptEIA kit (BD) in accordance with the manufacturer’s instructions. Assays were duplicated or triplicated and repeated three times at least. Tukey’s multiple comparison test was performed and P values less than 0.05 were considered as statistically significant.
